# Atypical Resting-State Functional Connectivity of Intra/Inter-Sensory Networks Is Related to Symptom Severity in Young Boys With Autism Spectrum Disorder

**DOI:** 10.3389/fphys.2021.626338

**Published:** 2021-03-24

**Authors:** Jia Wang, Xiaomin Wang, Runshi Wang, Xujun Duan, Heng Chen, Changchun He, Jinhe Zhai, Lijie Wu, Huafu Chen

**Affiliations:** ^1^Department of Children’s and Adolescent Health, Public Health College of Harbin Medical University, Harbin, China; ^2^Pediatric Health Care Section, Ningbo Women & Children’s Hospital, Ningbo, China; ^3^Ministry of Education (MOE), Key Lab for NeuroInformation, The Clinical Hospital of Chengdu Brain Science Institute, University of Electronic Science and Technology of China, Chengdu, China; ^4^School of Life Sciences and Technology, University of Electronic Science and Technology of China, Chengdu, China; ^5^School of Medicine, Guizhou University, Guiyang, China

**Keywords:** autism spectrum disorder, resting-state functional magnetic imaging, rest state network, auditory network, sensorimotor network, visual network

## Abstract

Autism spectrum disorder (ASD) has been reported to have altered brain connectivity patterns in sensory networks, assessed using resting-state functional magnetic imaging (rs-fMRI). However, the results have been inconsistent. Herein, we aimed to systematically explore the interaction between brain sensory networks in 3–7-year-old boys with ASD (*N* = 29) using independent component analysis (ICA). Participants were matched for age, head motion, and handedness in the MRI scanner. We estimated the between-group differences in spatial patterns of the sensory resting-state networks (RSNs). Subsequently, the time series of each RSN were extracted from each participant’s preprocessed data and associated estimates of interaction strength between intra- and internetwork functional connectivity (FC) and symptom severity in children with ASD. The auditory network (AN), higher visual network (HVN), primary visual network (PVN), and sensorimotor network (SMN) were identified. Relative to TDs, individuals with ASD showed increased FC in the AN and SMN, respectively. Higher positive connectivity between the PVN and HVN in the ASD group was shown. The strength of such connections was associated with symptom severity. The current study might suggest that the abnormal connectivity patterns of the sensory network regions may underlie impaired higher-order multisensory integration in ASD children, and be associated with social impairments.

## Introduction

Autism spectrum disorder (ASD), a neurodevelopmental disability, is associated with deficits in social communication and social interactions as well as restricted, repetitive patterns of behaviors, interests, and/or activities (American Psychiatric Association, 2013). Recent estimates are that ASD affects 1/59 children aged 8 years old (Baio et al., 2018), which is higher than the prevalence of 1/68 children in 2012 (Christensen et al., 2016) and the estimates of 1/110 children in 2006 (Autism and Developmental Disabilities Monitoring Network Surveillance Year 2006 Principal Investigators, 2009). A recent search for the prevalence of ASD in China found that Jilin City has a similar prevalence of autism in China to that in the West, at approximately 1%, and the results from Shenzhen and Jiamusi cities are also in line with Western estimates (Sun et al., 2019).

Sensory processing differences have long been associated with ASD, and they have recently been added to the diagnostic criteria for the updated diagnostic criteria in the Diagnostic and Statistical Manual of Mental Disorders, 5th Edition (*DSM-V*) (American Psychiatric Association, 2013). The previous paper integrated the empirical literature on sensory processing in ASD from the last decade and suggested a high prevalence of sensory symptoms, with reports ranging from 60 to 96% of children with ASD exhibiting some degree of atypical responses to sensory stimuli (Schauder and Bennetto, 2016). Clinical studies of two have indicated that central nervous system factors play a prominent role in abnormal sensory processing in ASD (Brandwein et al., 2015; Hannant et al., 2016). Sensory processing is a very complex process involving the cooperation of different brain regions. Different sensory-related neurons, neuron clusters, or dynamic interactions between brain regions are integrated to form the sensory functional network of the brain. Linear independent component analysis (ICA), a blind source signal separation method, is used to analyze fMRI data. It can extract a series of temporally independent brain activity signals or spatially independent brain networks. In recent years, ICA has been widely used in brain network research and has achieved many important research results, especially in the default mode network (DMN) and saliency network (SN) (Green et al., 2016; Duan et al., 2017; Bi et al., 2018; He et al., 2018; Guo et al., 2019). In the study of sensory network level, ICA found that children with ASD showed decreased FC in the VN and increased FC in the motor network (Uddin et al., 2013a; Ahmadi et al., 2014). However, the recent research suggested that the RSNs with increased FC include the auditory network (AN) and sensory-motor network (SMN), and mixed FC in visual network (VN) (Bi et al., 2018).

Past studies with ASD children have focused on connectivity patterns in a single area, an interesting network. The signals in different brain regions might show a certain correlation in time series even though there may be no structural connections. The principle of functional integration believes that the function of the brain is achieved through the interaction of multiple brain regions (Aertsen et al., 1989), when one sensory brain region is activated, other sensory brain regions are simultaneously activated or weakened. Therefore, the connection patterns between different sensory brain regions or brain networks reflect higher-order multisensory integration to a certain extent. Individuals with ASD atypically recruit visual brain regions during processing of simple auditory stimuli (Jao Keehn et al., 2017). Rs-fMRI study showed that weaker connectivity between the primary visual cortex and sensorimotor regions in preschool-aged children was correlated with increased sensory hypersensitivity in the visual/auditory domain (Shen et al., 2016). In the study of sensory network, Ahmadi et al. showed that in individuals with ASD, FC levels between the insular-temporal/ACC and the VN, DMN, and ACC were decreased; however, FC between the DMN and VN was increased (Ahmadi et al., 2014). Nebel et al. (2016) found increased FC between the VN and motor network in ASD school-age children. Although there has been a degree of progress in establishing the sensory FC architecture in RSNs in individuals with ASD, a consistent conclusion has not been reached, especially with a lack of evidence in younger ASD children.

As ASD is an early-onset disorder, children with ASD showed early brain overgrowth in the early stages of life (Courchesne et al., 2001; Sparks et al., 2002), and among autistic boys, 7-year-olds had the largest whole brain volume (Courchesne et al., 2001). In the study of fMRI, Nomi et al. found that children with ASD aged 11 and under, compared with age-matched TD children, exhibited hyperconnectivity within large-scale brain networks (Nomi and Uddin, 2015). Uddin et al. used rs-fMRI and found that hyperconnectivity may be more characteristic of young children with ASD, while hypoconnectivity may begin to emerge in adolescence and persist into adulthood (Uddin et al., 2013b). These previous mixed hyper- and hypoconnectivity results suggest that brain FC presents periodical features with increased age. According to the findings of structural and functional imaging, we focused on a narrow age range of 3–7 years in order to understand the neurobiological changes. Besides, we limited the study to male children to minimize developmental differences between different age groups and sexes (Carper et al., 2002; Bloss and Courchesne, 2007). The current study was motivated to examine sensory RSN dysregulation. We hypothesize that the children with ASD would exhibit atypical FC pattern involving inter/intra-sensory networks assessed using ICA. Sensory processing impairment, and associated brain abnormalities, have been shown to be highly correlated with patient outcome in social/emotional functioning, cognitive abilities (Corina and Singleton, 2009). We further tested whether atypical FC pattern changes of ASD could be a source of the abnormal sensory behavior and social impairments and repetitive patterns of behavior observed in children with ASD.

## Materials and Methods

### Participants

In total, 29 male children with ASD were recruited from the Children Development and Behavioral Research Center of Harbin Medical University, and 29 age-, resident-, and handedness-matched male TDs were recruited from local kindergartens; all the children in the two groups were composed of Han nationality. We carried out *t*-tests on the age, resident, and Mean FD_scrubbingg, and Chi-square test on the handedness to analyze the differences between two groups. The above factors of the two groups matched each other (Table 1). None of the children had a reported history of any severe medical problem or any neurological or psychiatric condition (with the exception of ASD), and none of the children were taking psychotropic medications. Written informed consent was obtained from the guardians of each subject prior to examination. This study was approved by the ethics review committee of Harbin Medical University.

**TABLE 1:** Demographic data (means ± SD) of ASD and TD group.

	ASD (*n* = 29)	TD (*n* = 29)	t/χ^2^	*p*
Age(years,x ± s)	4.93 ± 1.30	4.99 ± 1.01	−0.206^1^	0.837
Resident(city/rural)	26/3	23/6	1.184	0.277
Nation(Han nationality)	29	29	N/A	N/A
Handiness(right/left)	22/7	26/3	1.933^2^	0.164
PPVT(x ± s)	61.24 ± 17.09	99.69 ± 26.93	−6.492^1^	0.000*
Mean FD_scrubbing	0.129 ± 0.045	0.136 ± 0.037	−0.707^1^	0.483
AQ total score	84.19 ± 1.56	N/A	N/A	N/A
**AQ sub-scale**				
Social skills	16.42 ± 4.41	N/A	N/A	N/A
Attention switching	15.38 ± 3.98	N/A	N/A	N/A
Attention to detail	15.62 ± 6.26	N/A	N/A	N/A
Communication	19.31 ± 4.66	N/A	N/A	N/A
Imagination	17.46 ± 3.69	N/A	N/A	N/A
**ADOS sub-scale**				
Communication	6.15 ± 1.79	N/A	N/A	N/A
Social interaction	9.50 ± 2.30	N/A	N/A	N/A
Communication + social interaction	15.65 ± 3.54	N/A	N/A	N/A
Stereotyped behaviors and restricted interests	2.11 ± 5.19	N/A	N/A	N/A
**ADIR sub-scale**				
Social interaction	22.24 ± 3.53	N/A	N/A	N/A
Communication	15.00 ± 4.31	N/A	N/A	N/A
Restricted, repetitive, and stereotyped behaviors	7.00 ± 2.55	N/A	N/A	N/A
SRS total score	88.74 ± 2.06	N/A	N/A	N/A
**SRS sub-scale**				
Social perception	4.89 ± 6.08	N/A	N/A	N/A
Social cognition	18.85 ± 4.94	N/A	N/A	N/A
Social communication	34.22 ± 8.39	N/A	N/A	N/A
Social motivation	15.48 ± 4.72	N/A	N/A	N/A
Autism behavior pattern	15.30 ± 5.33	N/A	N/A	N/A
SSP total scale	147.82 ± 20.63	N/A	N/A	N/A
**SSP sub-scale**				
Tactile sensitivity	30.91 ± 4.12	N/A	N/A	N/A
Taste/Smell sensitivity	17.27 ± 2.84	N/A	N/A	N/A
Movement sensitivity	12.00 ± 2.79	N/A	N/A	N/A
Underresponsive/Seeks sensation	27.64 ± 4.37	N/A	N/A	N/A
Auditory filtering	18.55 ± 4.57	N/A	N/A	N/A
Low energy/weak	22.50 ± 5.57	N/A	N/A	N/A
Visual/Auditory sensitivity	18.96 ± 4.12	N/A	N/A	N/A

*ASD, autism spectrum disorder; TD, typical developing; PPVT, Peabody Picture Vocabulary Test; ADOS, Autism Diagnostic Observation Schedule; ADI-R, Autism Diagnostic Interview-Revised; N/A, not applicable (TDs did not receive the AQ, SSP, SRS, ADOS, and ADI-R).*

*^1^indicates t-test;*

^2^indicates chi-square test;* indicates statistical significance p < 0.001.

### Diagnosis and Clinical Assessment

The ASD diagnosis was based on the *DSM-5* (American Psychiatric Association, 2013) combined with the Autism Diagnostic Interview-Revised (ADI-R) (Lord et al., 1994) and Autism Diagnostic Observation Schedule (ADOS) (Lord et al., 2000). In addition, Peabody Picture Vocabulary Test (PPVT) is used as an estimation method for all children’s cognitive abilities (Dunn et al., 2006). The subjects with ASD were assessed via the Short Sensory Profile (SSP) (McIntosh et al., 1999), a useful screening instrument that is commonly used by occupational therapists to evaluate sensory processing difficulties in children. The Social Responsiveness Scale (SRS) (Constantino, 2002) was also applied to estimate their ability to engage in emotionally appropriate social interactions. The Autism Spectrum Quotient Children’s Version (AQ-child) (Baron-Cohen et al., 2001) is suitable for screening 4–11-year-old children with autism. The items in this scale were divided into 5 dimensions, including social skills, attention switching, attention to detail, communication, and imagination. Each item was scored with 0–3 points and 4 grades. The total score on the scale was 0–150. The higher the total score was, the more serious the autistic symptoms were.

### Neuroimaging Acquisition

The rs-fMRI data were collected using a 3.0 Tesla Achieva Magnetic Resonance System (Philips, The Netherlands) at the Department of MR Diagnosis of the affiliated hospital of Harbin Medical University. All MRI scans were performed under sedation using 50 mg/kg chloral hydrate following a strict clinical protocol. Rs-fMRI images were acquired with a gradient-echo echo-planar pulse: repetition time (TR) = 2,000 ms, echo time (TE) = 30 ms, 39 axial slices, flip angle = 90°, slice thickness/gap = 3.0 mm/1.0 mm, field of view = 240 × 240 mm^2^, and voxel size = 3.75 × 3.75 × 4 mm^3^. A total of 210 volumes (7 min) were obtained for each participant.

### fMRI Data Preprocessing

Data preprocessing was conducted with the Data Processing Assistant for Resting-State fMRI toolbox (DPARSF advanced edition v4.3)^1^ (Chao-Gan and Yu-Feng, 2010). The steps included the following: (1) removing the first 10 images to ensure steady-state longitudinal magnetization; (2) slice timing correction; (3) head motion correction with 6 rigid parameters (maximum head motion was less than 2 mm and 2 degrees for all children); (4) normalization to warp images into standard Montreal Neurological Institute (MNI) space at the resolution of 3 × 3 × 3 mm^3^; and (5) smoothing with a Gaussian kernel [full-width at half-maximum (FWHM) = 6 mm] to avoid introducing artificial local spatial correlations.

### Independent Component Analysis (ICA)

ICA was performed with the Group ICA application of the fMRI Toolbox (GIFTv3.0)^2^ (Calhoun et al., 2001). The total number of independent components (ICs) was identified by a preliminary dimension, which was estimated in accordance with the minimum description length criterion (Li et al., 2007). There were three further stages: (1) reducing the data dimension by principal component analysis; (2) estimating ICs by using the infomax algorithm; and (3) reconstructing in reverse the IC time series and spatial map of individual subjects (Bell and Sejnowski, 1995; Calhoun et al., 2001). First, the components related to artifacts were discarded upon visual inspection of spatial patterns. Then, four components which are related to sensory networks were selected and represented to be functionally relevant RSN templates in the GIFT toolbox, which were used as provided by previous studies (Beckmann et al., 2005; Damoiseaux et al., 2006). The four components from all subjects were selected by the maximum spatial correlation between ICs and the corresponding template (Liao et al., 2010). In the current study, the four templates were the AN, SMN, primary visual network (PVN), and higher visual network (HVN).

### Intranetwork Connectivity Analysis

We obtained the spatial pattern of the RSNs, which were the spatial maps of each RSN, across all the subjects and assessed using a one-sample *t*-tests approach [*p* < 0.001, false discovery rate (FDR) corrected]. And with age, Mean FD_scrubbing, handedness, and PPVT scores as covariates. Then, between-group differences within each network were obtained using two-sample two-tailed *t*-tests (voxel level *p* < 0.001, cluster level *p* < 0.05, AlphaSim corrected), with comparisons between groups limited to the voxels within each corresponding RSN pattern.

### Internetwork Connectivity Analysis

To investigate between-group differences in the FC between different RSNs identified from ICA, we extracted the time series of each RSN from each participant’s preprocessed data, with age, FD, handedness, and PPVT scores as covariates. Then, Pearson correlation analysis was used to calculate the correlation coefficients (*r*) for the time series in each pair of four RSNs, and the value of *r* was normalized by using Fisher’s *r* to *z* transformation. For each participant, a 4 × 4 FC matrix was obtained. Using a two-sample two-tailed *t*-test approach, we compared the FC values between the ASD and TD groups. We used effect size to measure the strength of the relationship between the ASD and TD groups. The significance level was set at *p* < 0.05 (FDR corrected).

### Behavioral Correlations With Intra/Internetwork Connectivity in the ASD Group

The present study explored whether altered FC was associated with the severity of symptoms in ASD. Pearson correlation analysis was utilized to determine the relationship between the FC values of the atypical RSNs [controlling for the covariates, including age, mean frame displacement (FD), and handedness] and the ADI-R, ADOS, SSP, SRS, and AQ scores in the ASD group. A non-parametric Spearman’s rank correlation analysis was performed to assess the associations between FC values of the atypical RSNs and the SSP-level scores.

## Results

### Resting-State Networks

**FIGURE 1:**
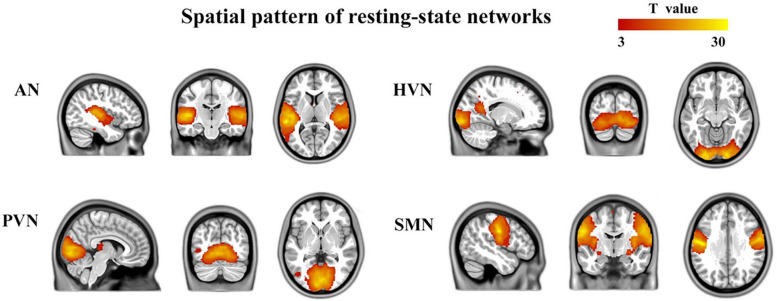
Four brain networks were determined by an independent component analysis. The color bar represents the *T-*value in each RSN (*p* < 0.001, FDR corrected). AN, auditory network; HVN, higher visual network; PVN, primary visual network; SMN, sensorimotor network

The number of ICs identified by ICA was 46. Four components were identified based on the highest spatial correlation with the RSN templates described previously, including auditory network (AN, 34th component, include bilateral superior temporal gyrus, bilateral middle temporal gyrus, left inferior temporal gyrus, bilateral caudate nucleus, left anterior cingulate and paracingulate gyri, and left precuneus), higher visual network (HVN, 40th component, include bilateral calcarine fissure and surrounding cortex, right lingual gyrus, right inferior occipital gyrus, left middle occipital gyrus, bilateral precuneus, right inferior frontal gyrus, opercular part, right middle frontal gyrus, right superior frontal gyrus, dorsolateral, right inferior frontal gyrus, triangular part, bilateral precentral gyrus, bilateral postcentral gyrus, right supramarginal gyrus, and left median cingulate and paracingulate gyri), primary visual network (PVN, 13th component, include bilateral lingual gyrus, bilateral calcarine fissure and surrounding cortex, left middle occipital gyrus, bilateral median cingulate and paracingulate gyri, left inferior parietal but supramarginal and angular gyri, left angular gyrus and left superior parietal gyrus), and sensorimotor network (SMN, 26th component, include bilateral cerebellum, bilateral precentral gyrus, bilateral postcentral gyrus, bilateral supplementary motor area, bilateral paracentral lobule). A typically spatial pattern in each RSN was obtained by using one-sample *t*-tests, which was consistent with previous studies (Liao et al., 2010; Ding et al., 2011; Zhang et al., 2015) (*p* < 0.001, FDR corrected) (Figure 1).

### Between-Group Differences in Intra-Network Connectivity

**FIGURE 2:**
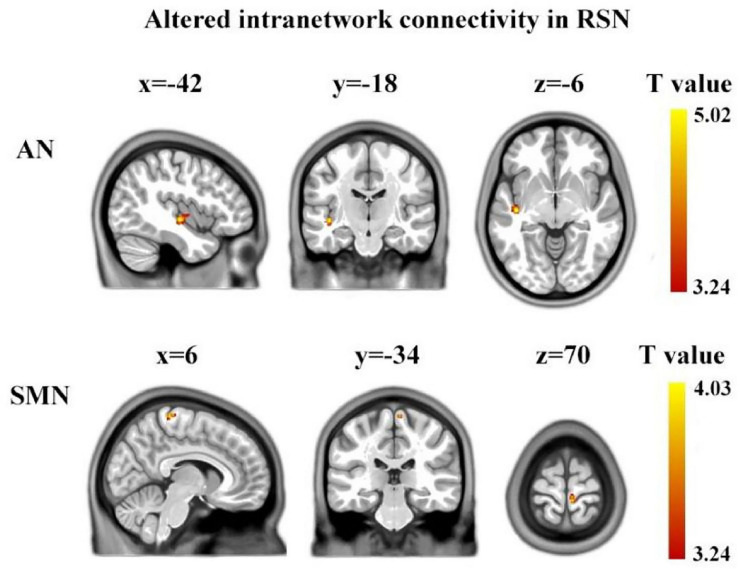
Brain regions showing between-group significant differences in intranetwork functional connectivity. The yellow color denotes the region with significantly increased connectivity in the ASD group, located in AN and SMN, respectively. The color bar represents the *T-*value (voxel level *p* < 0.001, cluster level *p* < 0.05, AlphaSim corrected). AN, auditory network; SMN, sensorimotor network.

Using two-sample two-tailed *t*-tests, we found the differences in intra-network connectivity between ASD group and TD group. Compared to TD, children with ASD showed significantly increased FC in the AN (left superior temporal gyrus, Brodmann’s area: 48, cluster size: 75, MNI: [−42, −18, −6]) and SMN (right paracentral lobule, Brodmann’s area: 4, cluster size: 27, MNI: [6, −34, 70]) (voxel level *p* < 0.001, cluster level *p* < 0.05 AlphaSim corrected) (Figure 2).

### Between-Group Differences in Inter-Network Connectivity

**FIGURE 3:**
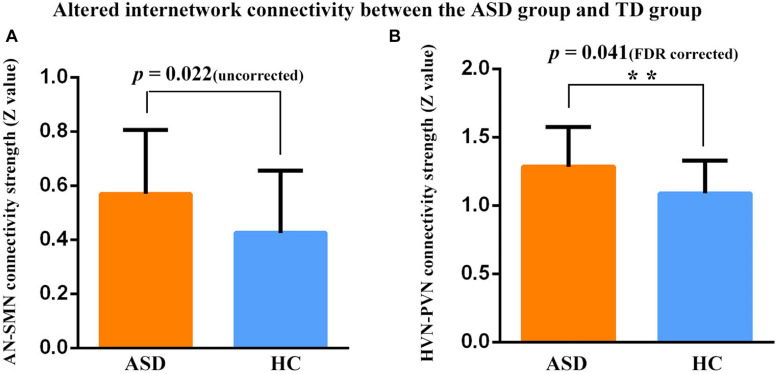
Connectivity strength (***z-***value) between two networks in two participant groups. **Represent significant differences after FDR correction. **(A)** Increased positive FC was found between the AN and SMN in the ASD group relative to the TD group (*p* = 0.022, uncorrected). **(B)** Increased positive FC was found between the PVN and HVN in the ASD group relative to the TD group (*p* = 0.041, FDR corrected). Error bars represent standard error of the mean.

Significant differences in FC patterns were found by comparing the correlation coefficients in the FC between ASD and TD groups. Compared to TDs, children with ASD showed significantly increased positive FC between AN and SMN (*p* = 0.022, uncorrected), PVN and HVN (*p* = 0.041, FDR corrected) (Figure 3).

### Relationship With ASD Symptoms

**FIGURE 4:**
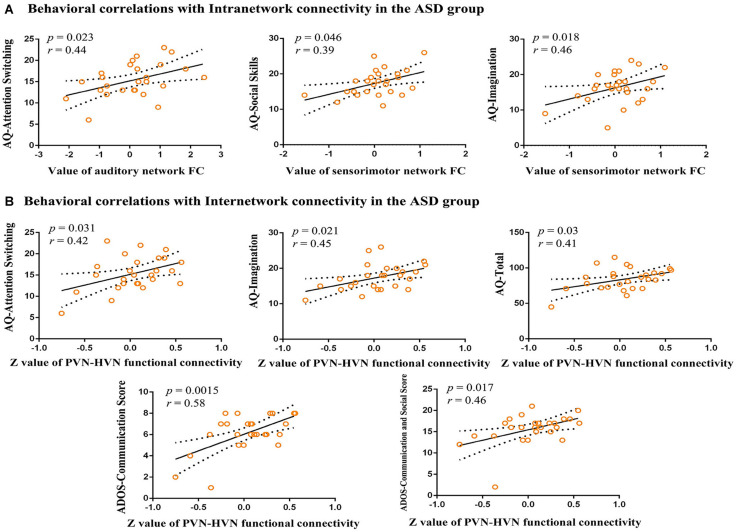
Correlations between functional connectivity and behavioral scores. **(A)** Behavioral correlations with intranetwork connectivity in the ASD group. Scatter plots show significant (*p* < 0.05) positive correlations between aberrant intranetwork connectivity and AQ scores. **(B)** Behavioral correlations with internetwork connectivity in the ASD group. Scatter plots show significant (*p* < 0.05) positive correlations between mean *Z-*values of aberrant internetwork connectivity and AQ scores and ADOS scores. AQ, Autism Spectrum Quotient Children’s Version; FC, functional connectivity; ADOS, Autism Diagnostic Observation Schedule.

In our study, we explore the relationship between aberrant intra-network connectivity and the AQ scores of children with ASD. The results showed significant (*p* < 0.05) positive correlations between the increased auditory network connectivity and the attention switching subscale of AQ. The social skill subscale and imagination subscales of AQ in children with ASD showed a significant positive correlation with increased sensorimotor network connectivity (Figure 4A). In addition, the aberrant inter-network connectivity between higher visual network and primary visual network showed significant positive correlations with attention switching subscale, imagination subscale and total score of AQ, as well as language and communication, communication, and social interaction subscales of ADOS (Figure 4B).

## Discussion

The present study found significant differences in FC at the network level between ASD boys aged 3–7 years and matched TDs. First, functional images revealed individuals with ASD showed increased FC in the AN and SMN, respectively. Second, when performing an internetwork functional connection analysis, we found significantly higher positive connectivity between the PVN and HVN in the ASD group. Third, the strength of such connections was associated with clinical scale scores, further demonstrating the robustness of our findings.

### Altered Intranetwork FC in ASD Children

We found that the left superior temporal gyrus (STG) in the AN had an excessive level of FC. Prior structural imaging studies had detected increased gray matter volume in the left STG across different life stages in ASD patients, including toddlers (Xiao et al., 2014), children (Retico et al., 2016), and adolescents (Lim et al., 2015). These findings in the left STG were in accordance with our previous structural neuroimaging study in 3–7-year-old ASD boys (Wang et al., 2017). A prominent view is that children with ASD develop excessive brain growth at 2–4 years of age (Courchesne et al., 2001; Sparks et al., 2002), and the development of the auditory cortex does not follow the normal growth epoch (Xiao et al., 2014; Lim et al., 2015; Retico et al., 2016). The theory of synaptic pruning proposed that there was a decreased synaptic pruning in autistic children and adolescents in the auditory area (Tang et al., 2014). With combined excessive brain growth and decreased synaptic pruning, children with ASD may have redundant synaptic connections in the immature auditory cortex. Nomi and Uddin (2015) further reported that changes in synaptic density may be related to changes in FC. The above findings further provided reliable evidence for increased FC in the auditory area. Correlation analysis with clinical scales found the excessive connectivity in the AN in children with ASD was positively correlated with attention switching of AQ (*r* = 0.44, *p* = 0.023). The correlation reflected that the excessive connections and increased energy consumption in the AN might affect the children with ASD cannot effectively process the auditory information, and show a certain disruption in the ability to switch attention such that “I frequently get so strongly absorbed in one thing that I lose sight of other things,” “In a social group, I can easily keep track of several different people’s conversations” (Baron-Cohen et al., 2001).

Our study reported that the children with ASD had a significantly stronger FC pattern in the right paracentral lobule of the SMN. Within the sensorimotor brain region, there was greater FC in the bilateral precentral and postcentral gyri in the ASD group than the TD group (Uddin et al., 2013a). Atypical FC in the sensorimotor region of ASD patients has been repeatedly reported by previous studies, although with mixed findings (Mostofsky et al., 2009; Anderson et al., 2011; Uddin et al., 2013a). When using the functional “connectedness” method and recurrent-seek strategy in the early stages of life, it was also found that ASD children were overconnected in the sensorimotor brain area (Chen et al., 2018), indicating that the ASD children may have abnormal function in the SMN. Atypical brain anatomy and neurodevelopment will inevitably lead to functional changes, with the evidence from behavioral studies further providing a wide range of sensory-motor dysfunction in patients with ASD (Whyatt and Craig, 2013; Mosconi and Sweeney, 2015). In the current study, the hyperconnectivity of the paracentric lobules in the SMN of the ASD children was more prominent. We hypothesized that sensorimotor dysfunction in children with ASD may be associated with dysfunction of the paracentral lobule, combined with the overlapping pattern of activity in the paracentral lobules in the task state (You et al., 2013; Gu et al., 2015). Martino et al. suggested that the paracentral lobule may be associated with abnormalities in the sense of a bodily self that is encased in the body observed in autistic patients (Di Martino et al., 2009). Additionally, sensorimotor dysfunction may play a key role in the impairments in social function in ASD populations (Hannant et al., 2016). Interestingly, this hypothesis was confirmed in the exploration of further associations with the AQ scale; that is, the excessive connections in the SMN (paracentral lobule) of those with ASD was significantly positively correlated with social skills and imagination (*r* = 0.39, *p* = 0.046; *r* = 0.46, *p* = 0.018). The association between the two indicates that the paracentral lobule processes information contributing to social dysfunction in patients with ASD, and its internal abnormal connections may be related to the pathological mechanisms underlying ASD.

### Altered Internetwork FC in ASD Children

Autistic individuals often exhibit enhanced perceptual abilities when engaged in visual search, visual discrimination, and embedded figure detection tasks (Samson et al., 2012). A recent study showed that local and global visual perception was incomplete in young children with ASD (Jobs et al., 2018). Visual information is transmitted through the conduction pathway. The primary visual pathways included the P (the ventral stream) and M pathways (the dorsal stream) (Tobimatsu and Celesia, 2006; Yamasaki et al., 2013, 2014). Evidence from visual evoked potentials has shown that dysfunctional activity in the P-color pathway in ASD (Fujita et al., 2011). In addition to findings from electrophysiological methods, RSN research finds ASD individuals showed that hyper-connectivity in visual cortex at initial link step distances (Martinez et al., 2019). At the same time, in the research of more complex visual networks, children with ASD showed decreased FC in the VN (Ahmadi et al., 2014). However, the recent research suggested the RSNs with mixed FC pattern in VN (Bi et al., 2018). In addition to the overall exploration, the visual brain regions/networks are further divided into primary and higher visual brain regions/networks. In the study of visual brain regions, the findings of robust and extensive overconnectivity in the striate (primary visual cortex) and extrastriate visual cortex (higher visual cortex) was intriguing in view of potential local biases in visual perception, supported thus far mainly by findings from behavioral studies (Dakin and Frith, 2005; Mottron et al., 2006). In the study of visual brain networks, children with ASD showed increased connectivity within the occipital pole network and decreased connectivity within the lateral visual network (Xu et al., 2019). To our knowledge, only a few rs-fMRI studies have examined FC between primary visual and higher visual regions in young children with ASD. When performing internetwork functional connection analyses, we found significantly higher positive connectivity between the PVN and HVN in the ASD group, even though we did not find abnormal FC in the ASD children in the PVN or HVN, respectively. The aberrant connection patterns were positively correlated with attention switching, imagination, and total score of the AQ scale and abnormal communication in the ADOS (*r* = 0.42, *p* = 0.031; *r* = 0.45, *p* = 0.021; *r* = 0.41, *p* = 0.03; *r* = 0.58, *p* = 0.0015, respectively). We hypothesize that the brain may not reasonably select and distinguish effective information and further communicate with higher visual networks, and these might promote children with ASD to pay too much attention to primary visual signals, which is associated with dysfunction of communication.

## Limitation

Our study had some limitations. First, our sample in this study was small and limited to young male children. It was worth exploring whether the research results are generalized to adolescents, adults, and female ASD patients. Second, future studies will further expand the sample and divide it into different subgroups according to the different types and degrees of abnormal sensory behavior that emerged from ASD children, and explore pertinently the relationship between the abnormal brain function networks related to sensory and specific clinical characterization. Third, the current study did not mention or measure power, other characteristics of children including socioeconomic indicators. Fourth, intranetwork connectivity with networks outside primary sensory networks was not examined. Fifth, in this young age range, a pediatric template may be more appropriate. Sixth, the cognitive level assessment of samples by PPVT in this study may be easily influenced by their language level. Finally, owing to their younger age and severity of ASD symptoms, all children cannot finish their scanning without sedation, and it is difficult to represent all ASD groups. Thus, all subjects were under sedation using chloral hydrate.

## Data Availability Statement

The original contributions presented in the study are included in the article/supplementary material, further inquiries can be directed to the corresponding author/s.

## Ethics Statement

The studies involving human participants were reviewed and approved by Ethics Review Committee of Harbin Medical University (Approval no. 2013004). Written informed consent to participate in this study was provided by the participants’ legal guardian/next of kin.

## Author Contributions

JW conceived of the study, performed the data acquisition, and drafted and revised the manuscript. XW was involved in the writing and revision of the manuscript and contributed to the acquisition of clinical data. RW was responsible for the analysis and interpretation of imaging data and helped to draft the manuscript. XD and HeC revised it and made instructive recommendations. LW and HuC conceived of the study and helped to receive to revise the manuscript. All authors read and approved the final manuscript.

## Conflict of Interest

The authors declare that the research was conducted in the absence of any commercial or financial relationships that could be construed as a potential conflict of interest.

## Footnotes

1 http://rfmri.org/DPARSF

2 http://mialab.mrn.org/software/gift/index.html#

## References

[B1] Aertsen A. M. Gerstein G. L. Habib M. K. Palm G. (1989). Dynamics of neuronal firing correlation: modulation of “effective connectivity”. *J. Neurophysiol.* 61 900–917. 10.1152/jn.1989.61.5.900 2723733

[B2] Ahmadi S. M. M. Mohajeri N. Soltanian-zadeh H. (2014). “Connectivity abnormalities in autism spectrum disorder patients: a resting state fMRI study,” in *Proceedings of the 22nd Iranian Conference on Electrical Engineering (ICEE)*, Tehran, 1878–1882.

[B3] American Psychiatric Association (2013). *The Diagnostic and Statistical Manual of Mental Disorders: DSM 5.* Arlington, VA: American Psychiatric Publishing, 991.

[B4] Anderson J. S. Druzgal T. J. Alyson F. Dubray M. B. Nicholas L. Alexander A. L. (2011). Decreased interhemispheric functional connectivity in autism. *Cereb. Cortex* 21 1134–1146. 10.1093/cercor/bhq190 20943668 PMC3077433

[B5] Autism and Developmental Disabilities Monitoring Network Surveillance Year 2006 Principal Investigators (2009). Prevalence of autism spectrum disorders - autism and developmental disabilities monitoring network, United States, 2006. *MMWR Surveill. Summ.* 58 1–24. 10.1016/s1871-1294(06)80002-3 20023608

[B6] Baio J. Wiggins L. Christensen D. L. Maenner M. J. Daniels J. Warren Z. (2018). Prevalence of autism spectrum disorder among children aged 8 years - autism and developmental disabilities monitoring network, 11 sites, United States, 2014. *MMWR Surveill. Summ.* 67 1–23. 10.15585/mmwr.ss6802a1 29701730 PMC5919599

[B7] Baron-Cohen S. Wheelwright S. Skinner R. Martin J. Clubley E. (2001). The autism-spectrum quotient (AQ): evidence from Asperger syndrome/high-functioning autism, males and females, scientists and mathematicians. *J. Autism Dev. Disord.* 31 5–17. 11439754 10.1023/a:1005653411471

[B8] Beckmann C. F. Deluca M. Devlin J. T. Smith S. M. (2005). Investigations into resting-state connectivity using independent component analysis. *Philos. Trans. R. Soc. Lond. Ser. B Biol. Sci.* 360 1001–1013. 10.1098/rstb.2005.1634 16087444 PMC1854918

[B9] Bell A. J. Sejnowski T. J. (1995). An information-maximization approach to blind separation and blind deconvolution. *Neural Comput.* 7 1129–1159. 10.1162/neco.1995.7.6.1129 7584893

[B10] Bi X. A. Zhao J. Xu Q. Sun Q. Wang Z. (2018). Abnormal functional connectivity of resting state network detection based on linear ICA analysis in autism spectrum disorder. *Front. Physiol.* 9:475. 10.3389/fphys.2018.00475 29867534 PMC5952255

[B11] Bloss C. S. Courchesne E. (2007). MRI neuroanatomy in young girls with autism: a preliminary study. *J. Am. Acad. Child Adolesc. Psychiatry* 46 515–523. 10.1097/chi.0b013e318030e28b 17420687

[B12] Brandwein A. B. Foxe J. J. Butler J. S. Frey H. P. Bates J. C. Shulman L. H. (2015). Neurophysiological indices of atypical auditory processing and multisensory integration are associated with symptom severity in autism. *J. Autism Dev. Disord.* 45 230–244. 10.1007/s10803-014-2212-9 25245785 PMC4289100

[B13] Calhoun V. D. Adali T. Pearlson G. D. Pekar J. J. (2001). A method for making group inferences from functional MRI data using independent component analysis. *Hum. Brain Mapp.* 14 140–151. 10.1002/hbm.1048 11559959 PMC6871952

[B14] Carper R. A. Moses P. Tigue Z. D. Courchesne E. (2002). Cerebral lobes in autism: early hyperplasia and abnormal age effects. *Neuroimage* 16 1038–1051. 10.1006/nimg.2002.1099 12202091

[B15] Chao-Gan Y. Yu-Feng Z. (2010). DPARSF: A MATLAB toolbox for “Pipeline” data analysis of resting-state fMRI. *Front. Syst. Neurosci.* 4:13. 10.3389/fnsys.2010.00013 20577591 PMC2889691

[B16] Chen H. Wang J. Uddin L. Q. Wang X. Guo X. Lu F. (2018). Aberrant functional connectivity of neural circuits associated with social and sensorimotor deficits in young children with autism spectrum disorder. *Autism Res.* 11 1643–1652. 10.1002/aur.2029 30475453 PMC6281874

[B17] Christensen D. L. Baio J. van Naarden Braun K. Bilder D. Charles J. Constantino J. N. (2016). Prevalence and characteristics of autism spectrum disorder among children aged 8 years–autism and developmental disabilities monitoring network, 11 sites, United States, 2012. *MMWR Surveill. Summ.* 65 1–23. 10.15585/mmwr.ss6503a1 PMC7909709 27031587

[B18] Constantino J. N. (2002). *The Social Responsiveness Scale.* Los Angeles, CA: Western Psychological Services.

[B19] Corina D. Singleton J. (2009). Developmental social cognitive neuroscience: insights from deafness. *Child Dev.* 80 952–967. 10.1111/j.1467-8624.2009.01310.x 19630887

[B20] Courchesne E. Karns C. M. Davis H. R. Ziccardi R. Carper R. A. Tigue Z. D. (2001). Unusual brain growth patterns in early life in patients with autistic disorder: an MRI study. *Neurology* 57 245–254. 10.1212/wnl.57.2.245 11468308

[B21] Dakin S. Frith U. (2005). Vagaries of visual perception in autism. *Neuron* 48 497–507. 10.1016/j.neuron.2005.10.018 16269366

[B22] Damoiseaux J. S. Rombouts S. A. Barkhof F. Scheltens P. Stam C. J. Smith S. M. (2006). Consistent resting-state networks across healthy subjects. *Proc. Natl. Acad. Sci. U.S.A.* 103 13848–13853. 10.1073/pnas.0601417103 16945915 PMC1564249

[B23] Ding J. R. Liao W. Zhang Z. Mantini D. Xu Q. Wu G. R. (2011). Topological fractionation of resting-state networks. *PLoS One* 6:e26596. 10.1371/journal.pone.0026596 22028917 PMC3197522

[B24] Duan X. Chen H. He C. Long Z. Guo X. Zhou Y. (2017). Resting-state functional under-connectivity within and between large-scale cortical networks across three low-frequency bands in adolescents with autism. *Prog. Neuropsychopharmacol. Biol. Psychiatry* 79(Pt B) 434–441. 10.1016/j.pnpbp.2017.07.027 28779909

[B25] Dunn L. Dunn L. M. Arribas D. (2006). *PPVT-III Peabody, Test de Vocabulario en Imágenes.* Madrid: TEA Ediciones.

[B26] Fujita T. Yamasaki T. Kamio Y. Hirose S. Tobimatsu S. (2011). Parvocellular pathway impairment in autism spectrum disorder: evidence from visual evoked potentials. *Res. Autism Spectr. Disord.* 5 277–285. 10.1016/j.rasd.2010.04.009

[B27] Green S. A. Hernandez L. Bookheimer S. Y. Dapretto M. (2016). Salience network connectivity in autism is related to brain and behavioral markers of sensory overresponsivity. *J. Am. Acad. Child Adolesc. Psychiatry* 55 618–626.e1. 27343889 10.1016/j.jaac.2016.04.013 PMC4924541

[B28] Gu X. Eilam-Stock T. Zhou T. Anagnostou E. Kolevzon A. Soorya L. (2015). Autonomic and brain responses associated with empathy deficits in autism spectrum disorder. *Hum. Brain Mapp.* 36 3323–3338. 10.1002/hbm.22840 25995134 PMC4545680

[B29] Guo X. Duan X. Suckling J. Chen H. Liao W. Cui Q. (2019). Partially impaired functional connectivity states between right anterior insula and default mode network in autism spectrum disorder. *Hum. Brain Mapp.* 40 1264–1275. 10.1002/hbm.24447 30367744 PMC6865537

[B30] Hannant P. Tavassoli T. Cassidy S. (2016). The role of sensorimotor difficulties in autism spectrum conditions. *Front. Neurol.* 7:124. 10.3389/fneur.2016.00124 27559329 PMC4978940

[B31] He C. Chen Y. Jian T. Chen H. Guo X. Wang J. (2018). Dynamic functional connectivity analysis reveals decreased variability of the default-mode network in developing autistic brain. *Autism Res.* 11 1479–1493. 10.1002/aur.2020 30270547

[B32] Jao Keehn R. J. Sanchez S. S. Stewart C. R. Zhao W. Grenesko-Stevens E. L. Keehn B. (2017). Impaired downregulation of visual cortex during auditory processing is associated with autism symptomatology in children and adolescents with autism spectrum disorder. *Autism Res.* 10 130–143. 10.1002/aur.1636 27205875 PMC5892834

[B33] Jobs E. N. Falck-Ytter T. Bölte S. (2018). Local and global visual processing in 3-year-olds with and without autism. *J. Autism Dev. Disord.* 48 2249–2257. 10.1007/s10803-018-3470-8 29411217 PMC5948270

[B34] Li Y. O. Adali T. Calhoun V. D. (2007). Estimating the number of independent components for functional magnetic resonance imaging data. *Hum. Brain Mapp.* 28 1251–1266. 10.1002/hbm.20359 17274023 PMC6871474

[B35] Liao W. Chen H. Feng Y. Mantini D. Gentili C. Pan Z. (2010). Selective aberrant functional connectivity of resting state networks in social anxiety disorder. *Neuroimage* 52 1549–1558. 10.1016/j.neuroimage.2010.05.010 20470894

[B36] Lim L. Chantiluke K. Cubillo A. I. Smith A. B. Simmons A. Mehta M. A. (2015). Disorder-specific grey matter deficits in attention deficit hyperactivity disorder relative to autism spectrum disorder. *Psychol. Med.* 45 965–976. 10.1017/s0033291714001974 25229248 PMC4413819

[B37] Lord C. Risi S. Lambrecht L. Cook E. H. Jr. Leventhal B. L. DiLavore P. C. (2000). The autism diagnostic observation schedule-generic: a standard measure of social and communication deficits associated with the spectrum of autism. *J. Autism Dev. Disord.* 30 205–223. 11055457

[B38] Lord C. Rutter M. Le Couteur A. (1994). Autism diagnostic interview-revised: a revised version of a diagnostic interview for caregivers of individuals with possible pervasive developmental disorders. *J. Autism Dev. Disord.* 24 659–685. 10.1007/bf02172145 7814313

[B39] Martinez K. Martinez-Garcia M. Marcos-Vidal L. Janssen J. Castellanos F. X. Pretus C. (2019). Sensory-to-cognitive systems integration is associated with clinical severity in autism spectrum disorder. *J. Am. Acad. Child Adolesc. Psychiatry* 59 422–433. 10.1016/j.jaac.2019.05.033 31260788

[B40] Di Martino A. Ross K. Uddin L. Q. Sklar A. B. Castellanos F. X. Milham M. P. (2009). Functional brain correlates of social and nonsocial processes in autism spectrum disorders: an activation likelihood estimation meta-analysis. *Biol. Psychiatry* 65 63–74. 18996505 10.1016/j.biopsych.2008.09.022 PMC2993772

[B41] McIntosh D. N. Miller L. J. Shyu V. Dunn W. (1999). “Overview of the short sensory profile,” in *Sensory Profile User’s Manual*, ed. Dunn W. (San Antonio, TX: The Psychologycal Corporation), 59–73.

[B42] Mosconi M. W. Sweeney J. A. (2015). Sensorimotor dysfunctions as primary features of autism spectrum disorders. *Sci. China Life Sci.* 58 1016–1023. 26335740 10.1007/s11427-015-4894-4 PMC5304941

[B43] Mostofsky S. H. Powell S. K. Simmonds D. J. Goldberg M. C. Caffo B. Pekar J. J. (2009). Decreased connectivity and cerebellar activity in autism during motor task performance. *Brain* 132(Pt 9) 2413–2425. 19389870 10.1093/brain/awp088 PMC2732264

[B44] Mottron L. Dawson M. Soulières I. Hubert B. Burack J. (2006). Enhanced perceptual functioning in autism: an update, and eight principles of autistic perception. *J. Autism Dev. Disord.* 36 27–43. 16453071 10.1007/s10803-005-0040-7

[B45] Nebel M. B. Eloyan A. Nettles C. A. Sweeney K. L. Ament K. Ward R. E. (2016). Intrinsic visual-motor synchrony correlates with social deficits in autism. *Biol. Psychiatry* 79 633–641. 26543004 10.1016/j.biopsych.2015.08.029 PMC4777671

[B46] Nomi J. S. Uddin L. Q. (2015). Developmental changes in large-scale network connectivity in autism. *Neuroimage Clin*. 7 732–741. 25844325 10.1016/j.nicl.2015.02.024 PMC4375789

[B47] Retico A. Giuliano A. Tancredi R. Cosenza A. Apicella F. Narzisi A. (2016). The effect of gender on the neuroanatomy of children with autism spectrum disorders: a support vector machine case-control study. *Mol. Autism* 7:5. 10.1186/s13229-015-0067-3 PMC4717545 26788282

[B48] Samson F. Mottron L. Soulières I. Zeffiro T. A. (2012). Enhanced visual functioning in autism: an ALE meta-analysis. *Hum. Brain Mapp*. 33 1553–1581. 21465627 10.1002/hbm.21307 PMC6870295

[B49] Schauder K. B. Bennetto L. (2016). Toward an interdisciplinary understanding of sensory dysfunction in autism spectrum disorder: an integration of the neural and symptom literatures. *Front. Neurosci.* 10:268. 10.3389/fnins.2016.00268 27378838 PMC4911400

[B50] Shen M. D. Li D. D. Keown C. L. Lee A. Johnson R. T. Angkustsiri K. (2016). Functional connectivity of the amygdala is disrupted in preschool-aged children with autism spectrum disorder. *J. Am. Acad. Child Adolesc. Psychiatry* 55 817–824. 27566123 10.1016/j.jaac.2016.05.020 PMC5003422

[B51] Sparks B. F. Friedman S. D. Shaw D. W. Aylward E. H. Echelard D. Artru A. A. (2002). Brain structural abnormalities in young children with autism spectrum disorder. *Neurology* 59 184–192. 12136055 10.1212/wnl.59.2.184

[B52] Sun X. Allison C. Wei L. Matthews F. E. Auyeung B. Wu Y. Y. (2019). Autism prevalence in China is comparable to Western prevalence. *Mol. Autism* 10:7. 10.1186/s13229-018-0246-0 PMC6394100 30858963

[B53] Tang G. Gudsnuk K. Kuo S. H. Cotrina M. L. Rosoklija G. Sosunov A. (2014). Loss of mTOR-dependent macroautophagy causes autistic-like synaptic pruning deficits. *Neuron* 83 1131–1143. 25155956 10.1016/j.neuron.2014.07.040 PMC4159743

[B54] Tobimatsu S. Celesia G. G. (2006). Studies of human visual pathophysiology with visual evoked potentials. *Clin. Neurophysiol.* 117 1414–1433. 16516551 10.1016/j.clinph.2006.01.004

[B55] Uddin L. Q. Supekar K. Lynch C. J. Khouzam A. Phillips J. Feinstein C. (2013a). Salience network-based classification and prediction of symptom severity in children with autism. *JAMA Psychiatry* 70 869–879. 23803651 10.1001/jamapsychiatry.2013.104 PMC3951904

[B56] Uddin L. Q. Supekar K. Menon V. (2013b). Reconceptualizing functional brain connectivity in autism from a developmental perspective. *Front. Hum. Neurosci.* 7:458. 10.3389/fnhum.2013.00458 23966925 PMC3735986

[B57] Wang J. Fu K. Chen L. Duan X. Guo X. Chen H. (2017). Increased gray matter volume and resting-state functional connectivity in somatosensory cortex and their relationship with autistic symptoms in young boys with autism spectrum disorder. *Front. Physiol.* 8:588. 10.3389/fphys.2017.00588 28861001 PMC5559537

[B58] Whyatt C. Craig C. (2013). Sensory-motor problems in autism. *Front. Integr. Neurosci.* 7:51. 10.3389/fnint.2013.00051 23882194 PMC3714545

[B59] Xiao Z. Qiu T. Ke X. Xiao X. Xiao T. Liang F. (2014). Autism spectrum disorder as early neurodevelopmental disorder: evidence from the brain imaging abnormalities in 2-3 years old toddlers. *J. Autism Dev. Disord.* 44 1633–1640. 24419870 10.1007/s10803-014-2033-x PMC4057630

[B60] Xu S. Li M. Yang C. Fang X. Ye M. Wei L. (2019). Altered functional connectivity in children with low-function autism spectrum disorders. *Front. Neurosci.* 13:806. 10.3389/fnins.2019.00806 31427923 PMC6688725

[B61] Yamasaki T. Maekawa T. Takahashi H. Fujita T. Kamio Y. Tobimatsu S. (2014). “Electrophysiology of visual and auditory perception in autism spectrum disorders,” in *Comprehensive Guide to Autism*, eds Patel V. Preedy V. Martin C. (New York, NY: Springer).

[B62] Yamasaki T. Fujita T. Kamio Y. Tobimatau S. (2013). Electrophysiological assessment of visual function in autism spectrum disorders. *Neurosci. Biomed. Eng.* 1 5–12.

[B63] You X. Norr M. Murphy E. Kuschner E. S. Bal E. Gaillard W. D. (2013). Atypical modulation of distant functional connectivity by cognitive state in children with autism spectrum disorders. *Front. Hum. Neurosci.* 7:482. 10.3389/fnhum.2013.00482 23986678 PMC3753572

[B64] Zhang Y. Liu F. Chen H. Li M. Duan X. Xie B. (2015). Intranetwork and internetwork functional connectivity alterations in post-traumatic stress disorder. *J. Affect. Disord.* 187 114–121. 26331685 10.1016/j.jad.2015.08.043

